# Quantification of Organ Motion in Male and Female Patients Undergoing Long-Course Radiation Therapy for Rectal Cancer in the Supine Position

**DOI:** 10.1016/j.adro.2022.101109

**Published:** 2022-10-21

**Authors:** Killian Nugent, Brian O'Neill, Victoria Brennan, Joann Lynch, Martin Higgins, Mary Dunne, Christina Skourou

**Affiliations:** St Luke's Radiation Oncology Network, Dublin, Ireland

## Abstract

**Purpose:**

Previous studies have reported data on the internal rectal motion of patients with rectal cancer treated in the prone position. With the introduction of intensity modulated techniques, more patients are treated in the more reproducible supine position. Data informing specific margins for this treatment position are sparse, as are data comparing rectal motion characteristics and factors in male and female patients. The purpose of this retrospective study was to quantify and compare the interfractional rectal movement characteristics of male and female patients with rectal cancer treated with long-course chemoradiation therapy in the supine position. The data will aid the generation of internal target volume margins accounting for this organ's internal physiological movements.

**Methods and Materials:**

Cone beam computed tomography (CBCT) images were acquired from 19 male and 16 female patients with rectal cancer on the first 3 days of treatment and weekly thereafter. The rectum, bladder, and femoral heads were delineated on the planning CT (PCT) and 6 CBCT for each patient. Overall, 245 images were analyzed. All patients were treated with a full bladder. The rectum was divided into three 5-cm segments (upper, mid, and lower). The motion of the rectum was quantified by documenting the anteroposterior and lateral distances as measured using fixed anatomic landmarks, namely from the anterior aspect of the sacrum and mid-left femoral head, respectively. These measurements were taken at 1-cm intervals from the inferior border of L5 vertebrae. The sigmoid was excluded from these measurements. Estimations of systematic and random physiological movement error were determined and margins were calculated.

**Results:**

Two hundred forty-five image sets (19 PCT + 114 CBCT for male, 16 PCT + 96 CBCT for female) on patients who had undergone long-course radiation therapy were analyzed. Rectal tumor location was 31% in the inferior rectum, 46% in the mid rectum, and 23% in the superior rectum. Random rectal motion (mean of the per-patient standard deviation [σ]) was largest for the upper and mid rectum in the anterior direction. There were statistically significant differences in σ between male and female patients in the left lateral motion of the mid and inferior rectum as well as the anterior, posterior, and right motion of the inferior rectum (mid left: *P* < .0005; lower left: *P* < .0005; lower posterior: *P* = .001; lower anterior: *P* = .032; lower right: *P* = .001). Suggested internal target volume margin guidelines are therefore nonisotropic and vary per segment of rectum and sex.

**Conclusions:**

In our present study, interfractional rectal motion is shown to be significantly different between male and female patients. Our data suggest that the use of asymmetrical sex-specific margins in patients with rectal cancer treated in the supine position should be considered.

## Introduction

Preoperative radiation therapy (RT) or chemo-RT (CRT) followed by total mesorectal excision is the internationally accepted standard of care in the management of locally advanced rectal cancer.^1^, ^2^, ^3^, ^4^ For rectal tumors that threaten the surgical circumferential resection margin and those with high-risk features, preoperative RT can improve the rates of complete surgical resection and reduce the rate of local recurrence. Delivery of radiation in the preoperative setting also reduces the rate of acute and late gastrointestinal (GI) toxicity compared with postoperative radiation^1^; therefore, knowledge of rectal motion characteristics with in situ tumor is required for accurate radiation planning.

There is a wealth of publications pertaining to normal rectal motion in prostate cancer treatment^5^, ^6^, ^7^ but with limited focus on rectal motion where the malignancy is rectal. Patients who are treated in the supine position have a more consistent bladder volume and reproducible setup in comparison to when treated in the prone position.^8^ Chong et al^9^ in 2011 reported data on internal rectal motion for patients with rectal cancer treated in the prone position^10^, ^11^, ^12^ in addition to producing margins to account for this geometric uncertainty. Further quantification of the interfractional mesorectal and rectal motion observed in patients with rectal cancer treated in the prone position has been published, but none of the studies have differentiated between male and female rectal motion characteristics. Data informing specific margins for the supine position are also sparse, as are data comparing rectal motion characteristics between male and female patients.

Intensity modulated RT (IMRT) compared with 3-dimensional conformal RT (3DRT) has been shown to achieve superior normal tissue avoidance (bladder and bowel [GI]) with comparable target dose coverage.^13^ Medical literature is lacking published randomized studies comparing the acute and late toxicities of preoperative 3DRT pelvic irradiation to IMRT. However, several small studies have reported considerable sparing of normal tissues using IMRT when compared retrospectively with 3DRT. Although 1 study by Hong et al^14^ did not show a reduction of GI toxicity with the use of IMRT, other retrospective clinical studies have demonstrated marked reductions in acute GI and genitourinary toxicity.^15^, ^16^, ^17^ Furthermore, in a meta-analysis by Wee et al,^18^ IMRT was shown to reduce grade ≥2 acute overall GI toxicity, diarrhea, and proctitis along with the incidence of grade 3 GI toxicity compared with 3DRT. Overall, there is evidence that IMRT use has steadily increased in the treatment of rectal cancer.^19^ With this increasing radiation conformity, steeper dose gradients, and consideration given to the option of a 2-phased approach to preoperative CRT plans as well as dose-escalation strategies, better understanding of organ motion characteristics is needed to generate accurate internal target volumes (ITVs).

The purpose of this retrospective study was to quantify and compare the interfractional rectal motion characteristics of male and female patients with rectal cancer treated with long-course CRT in the supine position. This information can then be used to generate ITV accounting for this organ's internal physiological movements.

## Methods and Materials

### Patient selection

Nineteen^20^ male patients and 16^20^ female patients who had undergone long-course CRT for locally advanced, histologically diagnosed rectal adenocarcinoma were retrospectively identified from our center's patient database. Patients were informed and written consent was obtained for the explicit use of their treatment data. All patients selected had undergone a pretreatment magnetic resonance imaging and had histologically confirmed rectal adenocarcinoma, with the following American Joint Committee on Cancer version VII staging^20^: cT3N0-2, cT4N0-2, cT(any)N1-2, cT(any)N(any) circumferential resection margin at-risk. All patients underwent a computed tomography (CT) thorax and abdomen with no evidence of metastatic disease. All patients had an Eastern Cooperative Oncology Group (ECOG) performance status of 0 to 2 and were age 18 years or older. The exclusion criteria for this study included: (1) previous RT to the pelvic region, (2) patients in whom induction chemotherapy was delivered before CRT, (3) history of inflammatory bowel disease, (4) previous hip replacement, (5) previous bowel surgery (excluding procedures/operations that would not result in small bowel adhesions), (6) patients with other syndromes/conditions associated with increased radio sensitivity, and (7) any other coexisting malignancies within the past 5 years other than nonmelanoma skin cancer. Patients who did not comply with the clinic's treatment protocol or who were not in a position to consent to this study were also excluded. After random selection from our database, cases were further excluded if a systemic error had been introduced at the time of planning CT (PCT) (rectum >6 cm on PCT due to gas/fecal matter). This study was approved by the St. Luke's Radiation Oncology Network Research Ethics Committee.

### PCT acquisition

Each patient underwent a planning contrast enhanced pelvic CT scan in the supine position. All patients were scanned with a full bladder as per local bladder-filling and hydration protocols (ie, 1.5 L recommended daily hydration with 3 cups of water 30 minutes before imaging) with bowel preparation/laxatives/enemas not used. Departmental dietary advice was given to reduce rectal gas. The scans were acquired from the top of L1 vertebra to 5-cm below the anal marker, with slice separation of 2.5 mm.

### Image acquisition and assessment

Kilovoltage cone beam CT (CBCT) images were acquired using a Varian iX LINAC with on- board CBCT capabilities and analyzed with the accompanying Eclipse treatment planning system (Varian Medical Systems, Palo Alto, CA). For treatment setup verification, CBCTs were acquired on the first 3 days of treatment and weekly thereafter and matched to the bony anatomy according to local departmental protocol. The scan length for the CBCTs was 15 cm reconstructed in 2.5-mm slices, starting from the inferior border of L5/superior border of S1. They were automatically coregistered and evaluated for accuracy and manual adjustments were made when necessary. Overall, 245 image sets, averaging 6 CBCTs per patient (19 PCT + 114 CBCT for male, 16 PCT + 96 CBCT for female) were analyzed.

### Organ volume, movement, and dimensions

**Figure 1:**
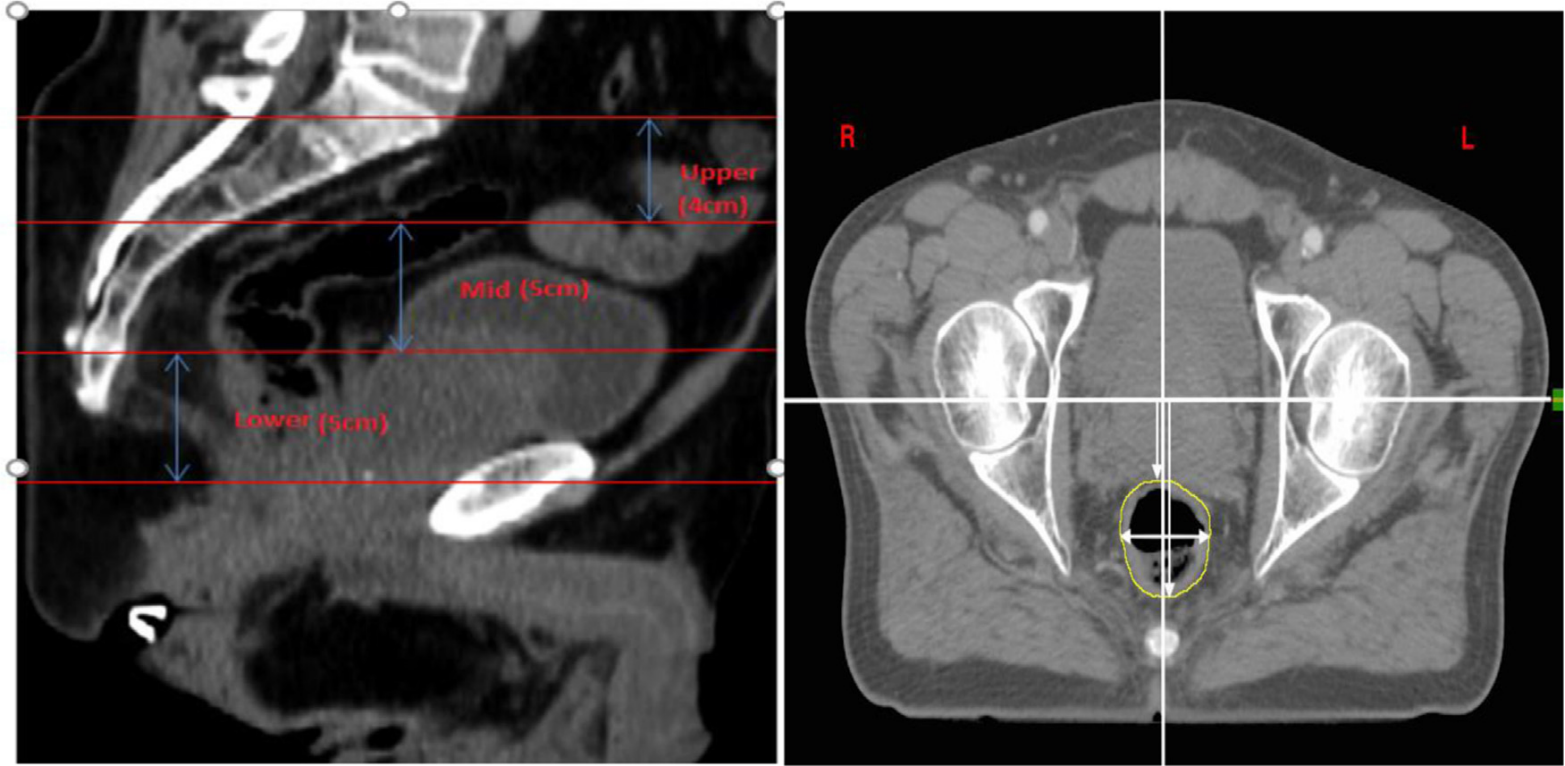
Left: Computed tomography (CT) image showing the upper, mid, and lower rectum as defined to be starting from the superior anterior border of S1. Right: Measurement of anterior, posterior, and lateral rectal wall motion. Anterior and posterior rectal movement was taken from the center of the left femoral head. Lateral rectal movement was taken from the center of the spine.

The site of disease varied along the rectum (Tables 1 and 2). The rectum, bladder, and femoral heads were delineated on the PCT and all CBCTs for each patient. To reduce uncertainties in rectal contouring, all PCT and CBCT contours were delineated by a single radiation oncologist and reviewed by a second. The rectum was divided into 3 segments (upper/sup 4 cm, mid 5 cm, and lower/inf 5 cm) as measured from the superior extent of the anterior S1 vertebrae. The volume of each segment and average diameter were acquired. The motion of the rectum was quantified by subtracting the difference in the location of each individual rectal wall between CBCT and PCT in the antero-posterior and lateral distances at set distant intervals as measured relative to a fixed axis on the axial images. The reference axis was derived by visually identifying the mid femoral head position and creating a horizontal line to bisect the femoral head and intersect an antero-posterior line drawn through the sacral midline (Fig. 1, left). These measurements were taken at 1-cm intervals from the superior extent of the anterior S1 vertebra. Each slice was described by an anterior, a posterior, and 2 lateral distances. Slices in the sigmoid or the anal canal were not included. The mean rectal wall displacement from all slices in each segment (upper, mid, and low) was used to determine the per-patient mean. The population data were then used to estimate the systemic and random physiological movement error by using the Van Herk margin calculation formula.^21^

**Table 1:** Male patient characteristics

Male patient number	Age (y)	Tumor stage	Tumor location
1	60	T3 N2	Lower
2	73	T3 N1	Upper
3	68	T3 N2	Mid
4	40	T3 N0	Upper
5	67	T3 N3	Lower
6	74	T3 N2	Mid
7	51	T3 N2	Lower
8	55	T2 N1	Upper
9	58	T3 N2	Upper
10	58	T3 N2	Mid
11	69	T3 N2	Mid
12	75	T3 N2	Upper
13	64	T3 N3	Lower
14	46	T3 N2	Mid
15	61	T3 N3	Mid
16	74	T3 N1	Lower
17	66	T2 N2	Lower
18	70	T3 N1	Lower
19	72	T2 N1	Lower

**Table 2:** Female patient characteristics

Female			
patient number	Age (y)	Tumor stage	Tumor location
1	57	T3 N1	Lower
2	69	T3 N2	Mid
3	62	T3 N2	Mid
4	59	T2 N1	Lower
5	45	T3 N2	Upper
6	51	T3 N2	Mid
7	55	T2 N1	Mid
8	71	T3 N2	Mid
9	63	T3 N2	Mid
10	69	T2 N1	Lower
11	71	T3 N2	Upper
12	62	T3 N2	Mid
13	66	T3 N3	Mid
14	42	T3 N2	Mid
15	67	T3 N1	Upper
16	72	T2 N1	Mid

### Statistical analysis

Pearson's correlation coefficient was used to analyze the correlation between average bladder volume and average anterior motion of each rectal segment and to analyze correlation between motion in individual rectal segments, demonstrating whether the rectum moves in unison as is the assumption when applying isotropic margins. Where assumptions for Pearson's were violated (eg, presence of outliers), a nonparametric Spearman test was conducted. The results with respect to statistical significance closely agreed and the *P* value remained always <.05. This was completed for the entire data set and for male and female groups separately.

Independent-sample *t* tests were conducted to compare the differences in mean motion for male and female patients. Preliminary investigations were conducted to ensure there was no violation of the assumptions of normality, linearity, and homoscedasticity. All statistical tests were 2-sided and assessed for significance at the .05 level. Statistical analyses were carried out using SPSS, version 25 (IBM SPSS Statistics 25).

### Calculation of systematic and random error

The Van Herk equation (2.5 Σ+ 0.7 σ),^21^ which accounts for systematic (Σ) and random error (*σ*) was used to propose an organ-specific motion for the rectum during long-course CRT. Our population systematic error was the spatial displacement in centimeters of the rectal walls in each individual vertical segment of the rectum from fixed bony anatomy (mean of standard deviation [σ]), and our random error (σ) was the mean of mean spatial displacement in centimeters of the rectal walls in each individual vertical segment of the rectum from fixed bony anatomy.

## Results

**Figure 2:**
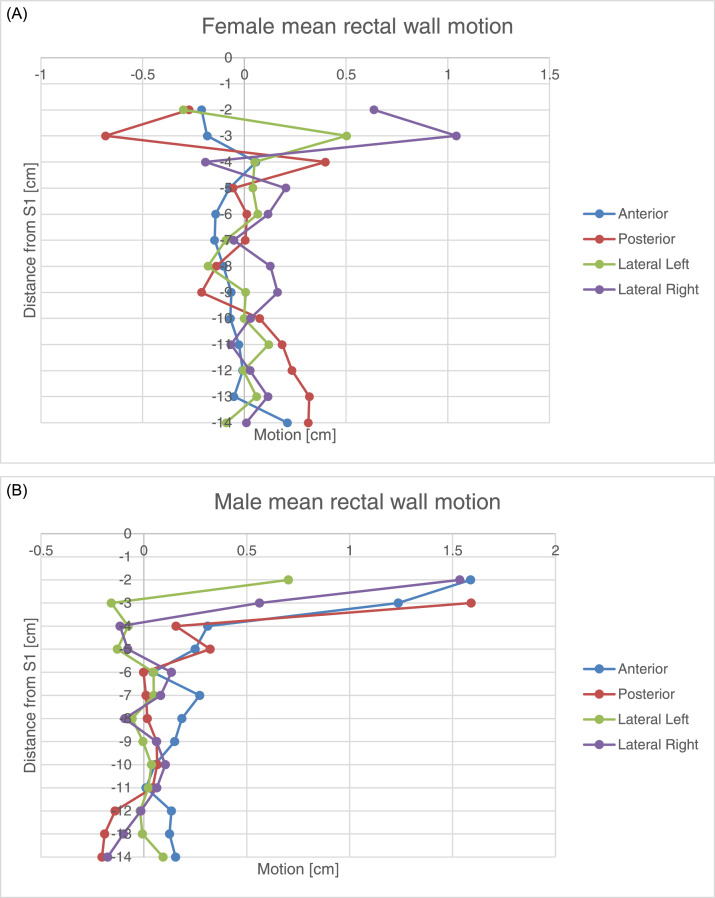
(A) Rectal wall movement (mean standard deviation of distance from bony landmarks) according to rectal location (female). (B) Rectal wall movement (mean standard deviation of distance from bony landmarks) according to rectal location (male).

Two hundred forty-five image data sets on patients who had undergone long-course chemotherapy were analyzed. The mean displacement for male and for female groups was plotted separately against the location of the displacement along the rectal wall (Fig. 2). The resulting parameters describing rectal wall motion are shown in Tables 3 and 4. For male and female patients combined, the random error as defined by Van Herk (σ) was largest in anterior direction for the upper and mid segment. For male patients only, σ was largest in the anterior direction for the upper segment and in the anterior direction for the mid rectal segment for female patients only.

**Table 3:** Change in rectal wall displacement and diameter from PCT according to upper, mid, or lower rectal location for male patients

**Upper (n = 7)**			**Mid (n = 19)**			**Lower (n = 19)**		
**Mean of SD (σ)**	**SD of SD**	**Mean of mean**	**Mean of SD (σ)**	**SD of SD**	**Mean of mean**	**Mean of SD (σ)**	**SD of SD**	**Mean of mean**

A 0.90	0.36	–0.07	0.72	0.24	0.09	0.52	0.12	0.06
P 0.58	0.24	0.00	0.42	0.13	–0.03	0.45	0.15	–0.09
L 0.55	0.13	–0.21	0.47	0.1	0	0.28	0.09	–0.02
R 0.57	0.18	–0.28	0.51	0.19	0.08	0.29	0.11	–0.02

*Abbreviations:* A = anterior; L = left; P = posterior; PCT = planning computed tomography; R = right; SD = standard deviation.

**Table 4:** Change in rectal wall displacement and diameter from PCT according to upper, mid, or lower rectal location for female patients

Upper (n = 10)			Mid (n = 16)			Lower (n = 16)		
Mean of SD (σ)	SD of SD	Mean of mean	Mean of SD (σ)	SD of SD	Mean of mean	Mean of SD (σ)	SD of SD	Mean of mean
A 0.69	0.15	–0.17	0.80	0.17	–0.06	0.61	0.12	0.27
P 0.47	0.16	–0.16	0.56	0.30	–0.1	0.65	0.17	–0.01
L 0.53	0.22	0.01	0.68	0.18	–0.05	0.47	0.15	0.05
R 0.58	0.35	0.14	0.64	0.20	0.09	0.53	0.23	0.01

*Abbreviations:* A = anterior; L = left; P = posterior; PCT = planning computed tomography; R = right; SD = standard deviation.

There were statistically significant differences in σ between male and female patients in the lateral directions in the mid and lower rectal segments as well as the anterior, posterior, and lateral motion in the lower rectal segment (mid L: *P* < .0005; lower L: *P* < .0005; lower P: *P* = .001; lower A: *P* = .032; lower R: *P* = .001).

The average anterior motion for each segment was found to be independent of the average bladder volume (ie, motion was not statistically correlated) for the combined male and female data. However, when female and male data were analyzed separately, there was a statistically significant correlation between the average bladder volume and the average posterior motion in the mid segment (*r* = 0.541, *P* = .031) for female patients and in the lower segment (*r* = 0.512, *P* = .025) for male patients.

Combining male and female data sets revealed a strong correlation between the anterior motion of the mid and lower segments (*r* = 0.501, *P* = .002) and a moderate correlation between lateral motion of the mid and lower segments (left: *r* = 0.376, *P* = .026; right: *r* = 0.653, *P* < .0005). In the combined data, these were the only statistically significant correlations between individual patterns of motion in the 3 segments.

When the female data were analyzed separately, only the anterior motion correlation between the mid and lower segments remained statistically significant (*r* = 0.637, *P* = .008).

However, in the male data set, there were statistically significant correlations between anterior, posterior, and lateral rectal wall motion of the mid and lower rectal segments (anterior motion of mid and lower rectum, *r* = 0.504, *P* = .028; posterior motion of mid and lower rectum, *r* = 0.459, *P* = .048; lateral motion of lower and mid rectum, left: *r* = 0.552, *P* = .014, right: *r* = 0.872, *P* ≤ .0005). In addition, the left lateral rectal motion of mid with upper rectal segments displayed a further statistically significant correlation (*r* = 0.894, *P* = .007).

The internal tumor volume (ITV) margins required to account for the anatomic motion of the rectum are shown in Tables 5 and 6. These margins exclude systematic and random errors from daily patient setup, as the displacements were referenced to individual anatomic markers (as described in the Methods and Materials section).

**Table 5:** Internal target volume margins for male patients

Direction	Upper (cm) n = 7	Mid (cm) n = 19	Lower (cm) n = 19
**Anterior**	2.47	1.49	1.22
**Posterior**	2.23	1.02	0.91
**Left**	1.61	1.09	0.87
**Right**	2.08	1.18	1.09

Internal target volume margins based on our data are nonisotropic and vary per segment of rectum and sex.

**Table 6:** Internal target volume margins for female patients

	Upper (cm)	Mid (cm)	Lower (cm)
Direction	n = 10	n = 16	n = 16
**Anterior**	1.93	1.69	1.57
**Posterior**	2.11	0.94	1.41
**Left**	1.44	1.47	1.04
**Right**	2.10	1.62	1.23

Internal target volume margins based on our data are nonisotropic and vary per segment of rectum and sex.

## Discussion

The main purpose of this retrospective study was to quantify rectal motion during supine rectal CRT and to investigate whether there are any deviations between male and female organ motion. This analysis is required for ITV derivation and therefore needed for the informed development of institutional planning target volume margins. We found the largest changes in motion during CRT to occur in the anterior mid and upper rectal segments in male patients and in the anterior, posterior, and right lateral motion in the upper rectal segment of female patients.

In the past, studies have focused on the motion on the mesorectum with others centering on the clinical target volume motion. Indeed, Ippolito et al^22^ have suggested that analyzing the motion of the mesorectal structure for rectal cancer is of significance as it is one of the first sites of tumor spread. Their analysis, akin to our rectal motion data, reported the largest mesorectal motion in the anterior upper third of the mesorectum. In this vein, the motion of the mesorectum as a whole during a course of preoperative RT has also been studied by both Brierley et al^10^ and Nijkamp et al.^23^ Brierley et al reported that the greatest mesorectal motion occurred in the anterior and posterior direction throughout mesorectal structure with the greatest movement occurring again in the upper third. Their study included 17 patients with stage II or III rectal cancer who underwent analysis of repeated CT planning scans at week 1, 3, and 5 of CRT. They used a biomechanical model-based deformable image registration technique to measure 3D spatial change in the rectum and reported mesorectal planning target volume margins as 4-mm right, 5-mm left, 7-mm anterior, and 6-mm posterior. Similarly, Nijkamp et al quantified the interfraction shape variation of the mesorectum for 28 patients treated with 5 fractions of 5 Gy in the supine position. Using pretreatment CBCT scans acquired daily, shape variation was quantified by the distance between the PCT and the CBCT delineations. In their study, the greatest motion of the mesorectum was seen in the superior and anterior direction (approximately 1.7 cm), with a large range of systematic (1.8 mm) and random (1.5 mm) shape variation found. Interestingly, they found comparable ranges for patients treated in the prone position. Tournel et al^12^ too evaluated the intrafractional motion of the mesorectum in 10 preoperative patients with rectal cancer by measuring maximum displacements of the mesorectum on selected interval axial slices on pre- and post-RT megavoltage CTs. This study added analysis of how the mesorectal tissue moved in the cranio-caudal direction, reporting mean shifts cranially of 3.2 mm (± 5.6 mm) and caudally of 3.2 mm (± 6.8 mm). In our study, we assumed that the anorectal junction is constant when accurate bone matching is used. Comparably to mesorectal motion, Nuyttens et al^24^ studied the motion of the clinical target volume in 10 patients with rectal cancer using serial CT scans during RT. Similar to Brierley et al, they found that the movement is greatest in the superior portion of the pelvis, and suggested margins should be adjusted depending on the position of the volume within the pelvis.

The behavior of rectal wall motion, however, as opposed to mesorectum alone, has become recently important with the current increased interest in single integrated boost,^25^ sequential boost, and boost dose escalation strategies in both preoperative CRT^26^ and in the setting of nonoperative management.^27^ Brierley et al also included analysis of rectal motion in their study and produced margins of 8-mm right, 8-mm left, 8-mm anterior, and 9-mm posterior. Interesting comparisons with the supine margins calculated in the current study can be made to the prone margins published by Chong et al,^9^ where a similar cohort of patients was investigated for setup in the prone position. Chong et al analyzed 123 CBCT data sets that were acquired from 16 patients undergoing CRT. The interfraction rectal motion, as in our study, was measured using fixed bony landmarks as reference to define the upper, mid, and lower rectum. Similar to our study, they found that the anterior rectal movements changed the most in the upper rectum followed by the mid rectum. Our margins were slightly larger for right and left rectal movement throughout each segment, particularly for our female data set compared with Chong et al's non–sex-specific margins (Table 7). Whether lateral rectal motion is not only influenced by dietary modifications, laxatives, and enemas but also the different effects of gravity between supine and prone setup positions is worth considering. The interfraction motion of a further subpart of the planning volume, rectal tumor itself, was studied by Kleijjen et al^28^ (patients in supine position) and by Brierley et al (patients in prone position). Kleijjen et al, using frequent magnetic resonance imaging to document rectal gross tumor volume movement, quoted weekly systematic and random errors in the range of 2.3 to 4.8 mm and 1.5 to 3.3 mm, requiring a margin of 1.43 cm to account for the upper range of gross tumor volume motion. Understanding both tumor and organ motion will be important for accurate conformal radiation.

**Table 7:** Internal target volume margins for a combined population

Direction	Upper (cm)	Mid (cm)	Lower (cm)
	n = 17	n = 35	n = 35
**Anterior**	2.12	1.58	1.41
**Posterior**	2.12	1.00	1.16
**Left**	1.73	1.27	0.95
**Right**	2.12	1.38	1.15

Internal target volume margins based on our data are nonisotropic and vary per segment of rectum and sex.

In addition, the current study highlights the potential differences in organ motion characteristics between male and female patients with rectal cancer undergoing CRT. There was no statistically significant correlation between rectal motion and bladder volume for the combined male and female data. This supports Chong et al's findings of a lack of significant correlation between rectal motion and bladder volume. However, there was a weak statistically significant correlation between the average bladder volume and the average posterior motion in the mid segment for female patients and in the lower segment for male patients when the sexes were analyzed separately. This may be due to the cushioning effect of the mesorectum acting as suspension for the anterior rectal wall against the compressive effects of bladder filling. Whether the physiological influence of dynamic bladder motion is mitigated by endometrial and prostate tissue could also be a factor. Nijkamp et al looked at the influence of the bladder on changes in the mesorectum and showed that the influence of bladder volume on mesorectum deformation was larger in female patients than in male, suggesting that the endometrium could have an influence on mesorectal motion. In our male population, there was a dependent relationship seen between the posterior and lateral motions of the mid and lower rectum not seen in our female population, as well as the lateral rectal motion of mid with upper rectal segments.

Indeed, for female patients, with the exception of the middle and lower anterior movements, the rectal segments moved independently of each other. The anatomic differences between male and female could account for the difference seen here. The compressing and distension effects of endometrial tissue on the longitudinal relationship of each of the individual rectal segments could account for this independence.

Further review of the statistical differences between the sexes reveals that in female patients the random motion error is higher. This partly accounts for the larger margins derived for female patients in the present study, with the greatest differences seen for the lower rectum. Sex differences were also noted by Nijkamp et al, who reported that although mesorectum random motion was similar for male and female patients, a systematic variation was 3-mm larger for female patients and needed to be accounted for by a larger margin. This increased variation suggests that the anatomic constraints of the lower bony pelvis are looser in women than in men and may require an increase in frequency of on-treatment imaging for female patients to counteract for this observed increase in random error.

The findings in the present study pertain to patients set up in the supine position. Similar to reported prone studies, the current findings also warrant nonisotropic margins. Of additional interest is the finding that in the supine setup male patients could have reduced margins in the mid and lower rectum while female patients may require larger lateral margins, suggesting that both setup position and sex have an influence of the motion characteristics of the rectum. Further studies to investigate sex-specific differences in rectal motion are warranted.

There are obvious practical challenges to adopting in practice asymmetrical sex-specific ITV margins, with increased target volume delineation time and training required to outline each individual rectal segment. Furthermore, our study suggests large margins are needed to account for motion in the upper segment (closer to the rectosigmoid junction), which may not be pragmatic to use. Indeed, our calculated margins needed to account for organ motion suggest a value for emerging daily adaptive RT techniques as an alternative to margins mandated for an entire treatment course.

## Conclusion

In the present study, rectal motion is shown to be significantly different between men and women as well as influenced by the treatment setup position. Our data suggest using asymmetrical sex-specific margins in all patients could be considered.

## Appendix Supplementary materials

Image, application 1
