# Supplementary material for: Quantification of Organ Motion in Male and Female Patients Undergoing Long-Course Radiation Therapy for Rectal Cancer in the Supine Position
Source: Adv Radiat Oncol. 2022 Oct 21;8(1):101109. doi: 10.1016/j.adro.2022.101109 (PMC9723314; doi:10.1016/j.adro.2022.101109)
Supplement: Supplementary file 1 [file mmc1.docx]

Appendix 1:

Target delineation and Radiotherapy Planning

The gross tumour volume (GTV T) outline referenced the macroscopic disease seen on the diagnostic MRI, endoscopy and clinician’s documented digital rectal exam (DRE). GTV nodes encompassed malignant nodes (mesorectal and pelvic side-wall nodes). Planning target volume (PTV 1) was created by expanding GTV T by 1cm in all dimension except in the mid and upper rectum where a 1.5cm expansion was used. Clinical target volume (CTV) nodes was generated from a 1cm expansion of GTV nodes and combined (CTV Comb) with an elective CTV (CTV E) which include the entire mesorectum and the internal iliac, presacral and obturator nodal regions with the external iliac nodal region included if tumour is cT4 or if inguinal nodes being contoured. PTV 2 was created from a 7mm expansion of CTV Comb with a final planning PTV being the combination of PTV 1 and PTV 2. 50.4Gy in 28 fractions was prescribed and delivered within a total of 5.5 weeks (treating Monday to Friday) and delivered with concurrent 5FU infusion dose of 225mg/m2/day for the duration of the treatment.
